# Unveiling the Metabolic Profile of First-Episode Drug-Naïve Schizophrenia Patients: Baseline Characteristics of a Longitudinal Study Among Han Chinese

**DOI:** 10.3389/fpsyt.2021.702720

**Published:** 2021-07-09

**Authors:** Qi Zhang, Hui He, Xia Bai, Liping Jiang, Wei Chen, Xiaoying Zeng, Yanjia Li, Antonio L. Teixeira, Jing Dai

**Affiliations:** ^1^The Clinical Hospital of Chengdu Brain Science Institute, MOE Key Lab for Neuroinformation, University of Electronic Science and Technology of China, Chengdu, China; ^2^School of Life Science and Technology, University of Electronic Science and Technology of China, Chengdu, China; ^3^Chengdu Forth People' s Hospital, Chengdu Mental Health Center, Chengdu, China; ^4^Neuropsychiatry Program, Department of Psychiatry and Behavioral Sciences, The University of Texas Health Science Center at Houston, Houston, TX, United States; ^5^Chengdu Medical College School of Nursing, Chengdu, China

**Keywords:** first-episode drug-naï ve schizophrenia, metabolic abnormalities, Homocysteine, triglyceride, prolactin

## Abstract

**Objective:** Metabolic and other medical conditions are frequently comorbid with schizophrenia. As they might be the side-effects of antipsychotic treatment, studying first-episode drug-naïve schizophrenia (FDSZ) provides a unique opportunity to investigate a direct pathogenic link between metabolic changes and schizophrenia. Here, we presented the methods and baseline unique metabolic profile of FDSZ patients without medical comorbidities unveiling subthreshold indices of metabolic disturbances.

**Method:** Drug-naïve individuals diagnosed with schizophrenia but without any previous medical conditions were invited to participate in the study. Participants were submitted to structured psychiatric and cognitive assessments, laboratory and neuroimaging tests. Subjects will be followed after antipsychotic treatment at 6, 24 and 48 weeks.

**Results:** During an 8-month-period, out of 103 patients presenting with first episode psychosis, 67 subjects (43.3% men, 56.7% women) were enrolled in the study. They had a mean ± SD age of 32.1 ± 8.7 years, with a mean BMI of 21.1 kg/m^2^ and 11.3 ± 3.6 years of schooling. Less than 1/3 reported a family history of mental illness. Upon laboratory assessment, 10.4%, 7.5%, and 11.9% of patients were identified with hyperhomocysteinemia, hypertriglyceridemia and hyperprolactinemia, respectively, with percentages of women relatively higher than men except for hypertriglyceridemia.

**Conclusions:** First episode schizophrenia patients, especially women, present subclinical metabolic abnormalities, independent of antipsychotic treatment.

## Introduction

Schizophrenia is a severe disabling psychiatric disorder marked by psychotic and negative symptoms alongside cognitive deficits. While its pathophysiology has not been fully elucidated, it is recognized that metabolic abnormalities (MAs) are frequent comorbid in these patients, contributing to their higher burden of medical diseases and complications. The average life expectancy of men and women with schizophrenia is, respectively, 15 and 12 years shorter than for those without schizophrenia (1), in which comorbidities of metabolic and cardiovascular diseases are widely recognized as major contributors (2). Actually, patients affected by schizophrenia are at increased risk for metabolic and cardiovascular diseases, including hypertension, type II diabetes mellitus, coronary artery disease (3, 4), in which various MAs have been described as playing an important role. Moreover, MAs have been associated with poorer functional outcomes (5), worse quality of life (6, 7), and non-compliance with antipsychotic therapy (8) in this group of individuals. Interestingly, previous studies showed that first-episode schizophrenia populations without or minimal antipsychotic exposure present with higher prevalence of MAs, including lipid profile disturbances, reduced uric acid levels, elevated homocysteine and prolactin levels (9–13). Until now, the underlying mechanism of increased subthreshold metabolic dysregulation in schizophrenia is still unclear.

Accumulating evidence indicates that complex interactions between sex and metabolism might affect the incidence of MAs in the schizophrenia population (14), and yet previously reported data on sex-specific association with MAs in schizophrenia are still controversial. Some reports demonstrate a higher prevalence of MAs among female patients (15–17), while other reports even show the opposite results (18), and others show no sex difference (19, 20). Many factors may lead to these contradictory results, such as antipsychotic treatment and previous medical comorbidities. It has long been recognized that taking antipsychotics increases the risk of MAs (21–24). Previous reviews and meta-analyses indicated that MAs increase after the initial exposure to antipsychotics in children and adolescents (25, 26) and in first-episode psychosis patients (27, 28). Accordingly, MAs have become a matter of concern in the early stages of antipsychotic treatment. For example, one recent real-world study by Tao Li et al. (29) revealed that insulin resistance and lipid metabolism disorders occurred as early as 2 weeks after the initiation of antipsychotic treatment in first-episode schizophrenia patients. However, considerable debate exists about whether schizophrenia itself can contribute directly to metabolic dysfunction. Distinguishing pre-existing risk from antipsychotic induced effects is important for the understanding of the basis of MAs in the context of schizophrenia. First episode and drug naïve (FEDN) patients without medical comorbidities provide a unique opportunity to minimize confounding factors and examine MAs in schizophrenia patients without antipsychotics. Moreover, sex-specific MAs in FEDN schizophrenia without medical comorbidities have not been fully investigated.

Therefore, we designed a research project in a naturalistic setting, the First-Episode Drug-Naïve Schizophrenia without Metabolic and Cardiovascular Diseases Study, to investigate metabolic problems that might increase cardiovascular risk in the early stage of schizophrenia. The aim of the current report is to present the methods and the baseline characteristics of subjects enrolled in the study, in order to understand whether schizophrenia itself is associated with MAs.

## Study Design

### Subjects

For identification of cases, the Chengdu Fourth People's Hospital (Chengdu Mental Health Center, CMHC) recruited patients between May 1, 2018, and Dec 31, 2018.

At the time of enrollment of the study, participants were between the age of 18 and 55 years, experiencing a first psychotic episode of schizophrenia. Participants had never taken antipsychotics before. All patients met the diagnostic criteria of schizophrenia, as defined in the International Classification of Diseases, Eleventh Edition (ICD-11) (30).

Exclusion criteria were: (1) concurrent diagnosis of psychiatric disorders defined in ICD-11 other than schizophrenia; (2) concurrent treatment with anti-diabetic or lipid-lowering agents or special diets to lower glucose or lipid levels, or use of immunosuppressive agents; (3) diagnosis of diabetes, dyslipidemia, or any endocrine disease; (4) ongoing infections or allergies, history of alcohol or other substance use, autoimmune disorders, pregnancy or breastfeeding, known medical conditions that might affect metabolism, and history of diabetes or lipid disorders.

**Figure 1:**
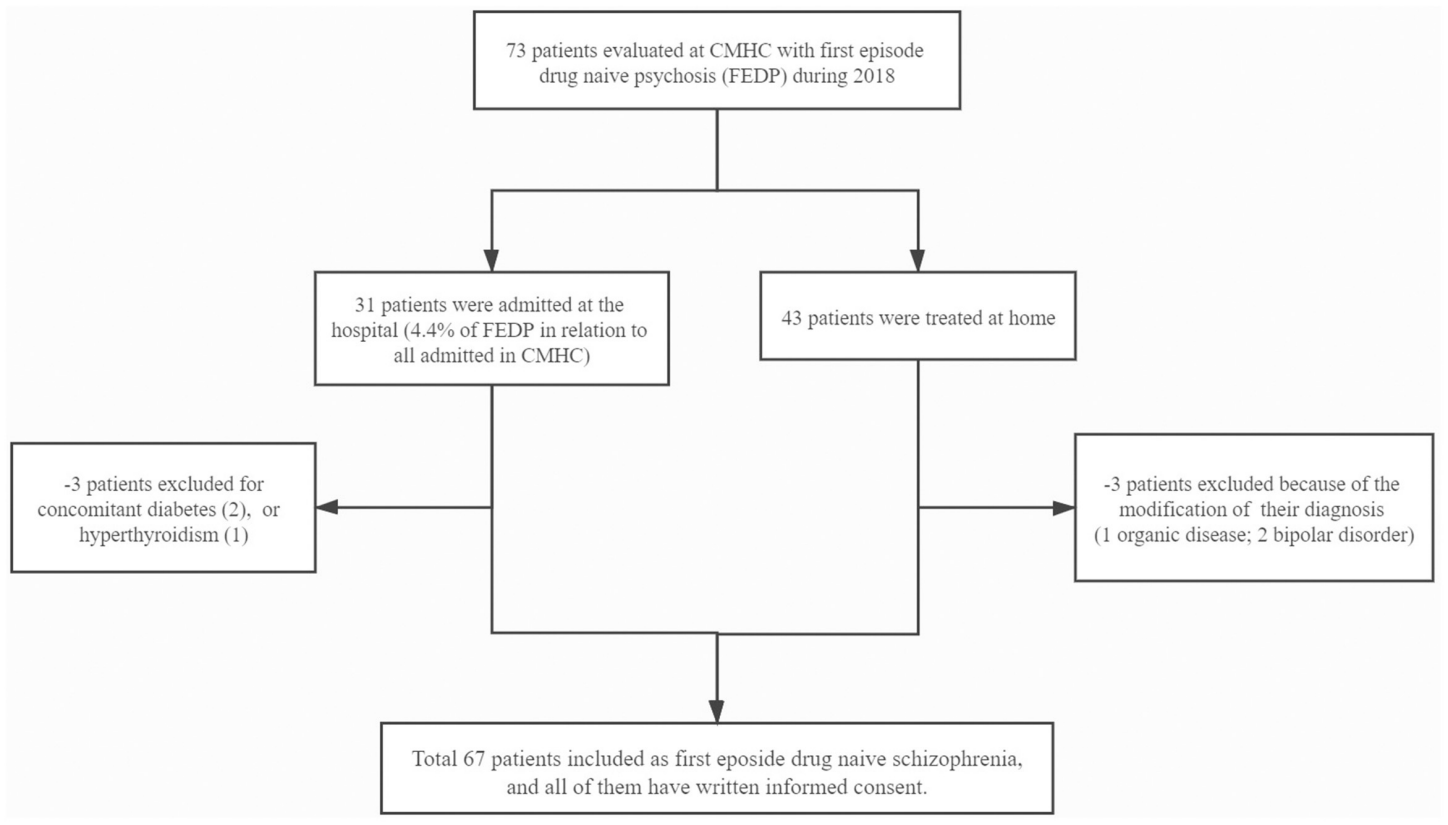
Flow chart of the inclusion and exclusion of patients (Chengdu, China, 2018).

The study was approved by the ethics committees of the West China Hospital of Sichuan University (ER136, 2017) and CMHC (ER15, 2017), as well as all subjects participated after providing a written, informed consent (See Figure 1).

### Methods

The study was conducted in four phases. On phase 1, two trained interviewers visited these participants and their families at CMHC, explained in detail the nature of the survey and invited them to participate. In the case of acceptance, all participant signed a written informed consent and was submitted to a detailed and structured evaluation that included: socio-demographic information, previous and current medical diagnoses, additional psychiatric medical information (medications in use with specific dose regimens, frequency of attendance to medical consultation), and general physical examination with weight and height, blood pressure and heart rate record. Positive and Negative Syndrome Scale (PANSS) (31) to assess schizophrenia symptoms; Hamilton Anxiety Scale (HAMA) (32) to assess anxiety; Hamilton Depression Scale-24 items (HAMD-24) (33) to assess depression. In addition, each subject was asked about suicide ideation, suicide plan, and suicide attempt, with items taken directly from the National Comorbidity Survey (34). These items were: “Have you ever seriously thought about committing suicide?” “Have you ever made a plan for committing suicide?” “Have you ever attempted suicide?” The clinical evaluation was conducted by team composed of four board certified psychiatrists, all of them with at least three years of experience in psychiatric assessment. Training sessions in order to standardize the clinical procedures were previously carried out by the group.

On phase 2, participants were submitted to a structured cognitive examination including: Brief Assessment of Cognition in Schizophrenia (BACS) (35) to evaluate the overall cognitive functioning; Wisconsin Card Sorting Test (WCST) (36) to evaluate executive functioning; Raven's Standard Progress Matrices (SPM) (37) to test the ability of observation and reasoning; Wechsler Adult Intelligence Scale-Revised (WAIS-R) (38) to measure the general intelligence abilities; and the Stroop Color and Word test (SCWT) (39) to test attention and executive functions. Neuropsychological and functional assessments were conducted by a team of three neuropsychologists, two psychologists and one occupational therapist, all with at least 3 years of clinical experience.

On phase 3, all participants were invited to provide a blood sample for laboratory tests, including uric acid (UA), homocysteine (HC), total cholesterol (TC), triglyceride (TG) and prolactin (PRL). Previous studies have reported that first-episode schizophrenia populations present with decreased UA levels, increased HC, TC, TG and PRL levels (9–13). Considering that a substantial body of evidence has accumulated that indicates hypouricemia (40, 41), hyperhomocysteinemia (42), hypercholesterolemia (43), hypertriglyceridemia (44) and hyperprolactinemia (45) are associated with cardiovascular risk, it was necessary to investigate hypouricemia, hyperhomocysteinemia, hypercholesterolemia, hypertriglyceridemia and hyperprolactinemia in these patients. As described in previous studies (46–50), hypouricemia was defined when UA concentration was <2.0 mg/dL; hyperhomocysteinemia, hypercholesterolemia, hypertriglyceridemia and hyperprolactinemia were defined when HC, TC, TG and PRL concentration exceeded 15.0 μmol/L, 200.0 mg/dL, 1.70 mmol/L, and 20.0 ng/mL, respectively. Blood samples were drawn after overnight fasting of at least 12 h between 06:00 to 07:00 a.m. Blood was sampled using anticoagulant-free tubes and kept for 1 h at 4 °C (for platelet activation) before serum was isolated (centrifugation at 3,000 rpm for 20 min at 4 °C). They also underwent neuroimaging test through a 3.0 Tesla SIEMENS Trio Tim scanner equipped with a 32-chanel head coil located at West China Hospital, Sichuan University, to measure volume T1& T2, DTI and rest-state functional MRI.

On phase 4, all patients are expected to be followed at 6, 24 and 48 weeks after enrollment. They will repeat clinical, neuropsychological and functional evaluations in each time point. In addition, all patients will be evaluated regarding clinical treatment outcomes, including: Clinical Global Impressions Scale (CGIS) (51), Rating Scale for Extrapyramidal Side Effects (RSESE) (52), Udvalg for Kliniske Undersogelser (UKU) (53), Barnes Akathisia Rating Scale (BARS) (54) and Abnormal Involuntary Movement Scale (AIMS) (55).

### Statistical Analysis

Descriptive tables and measures were used to establish a demographic, psychopathological and cognition-related profile of the patients enrolled in this study.

Group differences of the female and male were compared using one-way ANOVA for continuous variables and chi square for categorical variables. Since the scores of PANSS, HAMD-24 and HAMA were normally distributed in the male and female (Kolmogorov-Smirnov one-sample test; both *p* > 0.05), the principal outcome analysis consisted of one-way analysis of variance (ANOVA). Where there was a significance in ANOVA, the effect of age, education, smoking, and body mass index (BMI) were tested by adding these variables to the analysis model as covariates.

The PASW Statistics 18.0 software (SPSS Inc., Chicago, IL, USA) was used to do all statistical analysis. Data were presented as rate (%) or mean ± SD. All *p*-values were 2-tailed at the significant level of <0.05.

## Results

We evaluated 73 subjects presenting with first episode of psychosis at the CMHC from May 2018 to December 2018, of whom 31 needed to be hospitalized for further observation (Figure 1). Thirty six subjects were excluded from the study due to the following reasons: (1) two patients had diabetes and one had hyperthyroidism; (2) three had their psychiatric diagnosis changed (one organic disease, two bipolar disorder). Finally, we fully evaluated 67 individuals from Chengdu, Sichuan Province, China, being 38 (56.7%) women and 29 (43.3%) men, aged 21.1 ± 3.4 years, with educational level of 11.3 ± 3.6 years. Tables 1–3 depicted data on baseline general characteristics.

**Table 1:** Distribution of participants by demographics characteristics (Chengdu, China, 2018) by sex.

**Characteristics**		**Female (*N* = 38)**	**Male (*N* = 29)**	**F or *x*^**2**^**	**df**	***P***	**Total (*N* = 67)**
Age (years) *n* (%)	21–30	21 (55.3)	13 (44.8)	2.897	32	0.098	34 (50.7)
	31–40	5 (13.1)	13 (44.8)	0.647	16	0.433	18 (26.9)
	41–50	12 (31.6)	3 (10.4)	0.009	13	0.927	15 (22.4)
M (S.D.)	Total	32.4 (9.8)	31.8 (7.0)	0.070	65	0.793	32.1 (8.7)
BMI (kg/m^2^) M (S.D.)		20.7 (3.3)	21.7 (3.4)	1.479	65	0.228	21.1 (3.4)
Overweight (≥24.0 kg/m^2^) *n* (%)		6 (15.8)	6 (20.7)	0.028	11	0.871	12 (17.9)
Educational level (years) *n* (%)	<6	2 (5.3)	0 (0)				2 (3)
	6–9	13 (34.2)	11 (37.9)	2.828	22	0.107	24 (35.8)
	10–12	10 (26.3)	10 (34.5)	1.976	18	0.177	20 (29.9)
	13+	13 (34.2)	8 (27.6)	0.530	19	0.475	21 (31.3)
M (S.D.)	Total	11.2 (4.2)	11.5 (2.8)	0.131	65	0.178	11.3 (3.6)
Marital status *n* (%)	Unmarried	13 (34.2)	19 (65.5)				32 (47.8)
	Married	22 (57.9)	10 (34.5)				32 (47.8)
	Divorce	3 (7.9)	0 (0)				3 (4.4)
	Total			8.248	65	0.006	
Smoking *n* (%)	Yes	0 (0)	19 (65.5)				19 (28.4)
	No	38 (100)	10 (34.5)				48 (71.6)
	Total			70.045	65	<0.001	
Suicide ideation *n* (%)	Yes	10 (26.3)	3 (10.3)				13 (19.4)
	No	28 (73.7)	26 (89.7)				54 (80.6)
	Total			2.711	65	0.104	
Suicide plan *n* (%)	Yes	1 (2.6)	1 (3.5)				2 (3)
	No	37 (97.4)	28 (96.5)				65 (97)
	Total			0.037	65	0.849	
Suicide attempt *n* (%)	Yes	1 (2.6)	1 (3.5)				2 (3)
	No	37 (97.4)	28 (96.5)				65 (97)
	Total			0.037	65	0.849	

*BMI, body mass index; n, number; M, mean; S.D., standard deviation*.

**Table 2:** Mean baseline psycho-pathological scores for patients (Chengdu, China, 2018) by sex.

	**Female (*N* = 38)**	**Male (*N* = 29)**	***F***	**df**	***p***	**Total (*N* = 67)**
HAMD-24, total score, M (S.D.)	16.2 (9.1)	12.6 (7.5)	2.875	65	0.095	14.6 (8.6)
HAMA, total score, M (S.D.)	11.2 (7.1)	9.9 (7.0)	0.528	65	0.470	10.6 (7.0)
PANSS, total score, M (S.D.)	99.6 (13.3)	92.3 (10.4)	5.843	65	0.018	96.4 (12.6)
P subscore, M (S.D.)	25.8 (3.9)	25.2 (3.2)	0.380	65	0.540	25.5 (3.6)
N subscore, M (S.D.)	23.6 (5)	21.7 (4.7)	2.474	65	0.121	22.8 (4.9)
G subscore, M (S.D.)	50.2 (7.7)	45.4 (5.4)	8.166	65	0.006	48.2 (7.2)

*HAMD-24, hamilton depression scale-24; HAMA, hamilton anxiety scale; PANSS, positive and negative syndrome scale; P, positive symptoms; N, negative symptoms; G, general psychopathological symptoms; n, number; M, mean; S.D., standard deviation*.

**Table 3:** Mean baseline cognitive test scores for patients (Chengdu, China, 2018) by sex.

	**Female (*N* = 38)**	**Male (*N* = 29)**	***F***	**df**	***p***	**Total (*N* = 67)**
MQ, M (S.D.)	57.3 (30.1)	62.7 (30.9)	0.184	65	0.472	59.6 (30.3)
Verbal IQ, M (S.D.)	76.8 (33.0)	80.8 (32.1)	0.018	65	0.622	78.6 (32.4)
Performance IQ, M (S.D.)	73.3 (32.9)	79.1 (32.9)	0.021	65	0.478	75.9 (32.7)
Total IQ, M (S.D.)	75.6 (33.1)	79.4 (32.7)	0.015	65	0.638	77.2 (32.7)
**SCWT**						
RT of Test I, s, M (S.D.)	12.9 (9.3)	13.9 (8.6)	0.266	65	0.663	13.3 (9.0)
RT of Test II, s, M (S.D.)	20.9 (13.7)	30.3 (26.4)	3.216	65	0.065	25.0 (20.6)
RT of Test III, s, M (S.D.)	16.7 (14.5)	15.6 (10.5)	0.537	65	0.723	16.2 (12.8)
RT of Test IV, s, M (S.D.)	34.5 (22.4)	39.6 (25.8)	0.199	65	0.387	36.7 (23.9)
BACS, total score, M (S.D.)	15.8 (21.4)	26.7 (16.4)	2.274	65	0.026	20.5 (20.0)
Verbal memory, M (S.D.)	22.1 (20.7)	33.8 (13.2)	5.877	65	0.006	27.1 (18.7)
Digit sequencing, M (S.D.)	35.0 (13.5)	39.5 (13.5)	3.922	65	0.299	36.9 (17.6)
Token motor task, M (S.D.)	30.5 (20.0)	43.0 (14.4)	2.989	65	0.005	35.9 (18.5)
Verbal fluency, M (S.D.)	19.7 (8.7)	23.4 (8.4)	0.003	65	0.087	21.3 (8.7)
Symbol coding, M (S.D.)	23.4 (15.5)	30.6 (12.8)	1.514	65	0.045	26.5 (14.7)
Tower of london, M (S.D.)	37.6 (23.0)	47.6 (11.7)	10.659	65	0.024	41.9 (19.4)

*MQ, memory quotient; RT, reaction time; SCWT, stroop color and word test; BACS, brief assessment of cognition in schizophrenia; M, mean; S.D., standard deviation*.

As shown in Table 1, there was no significant difference in age (32.4 ± 9.8 years vs. 31.8 ± 7.0 years), education (11.2 ± 4.2 years vs. 11.5 ± 2.8 years), BMI (20.7 ± 3.3 kg/m^2^ vs. 21.7 ± 3.4 kg/m^2^), suicidal ideation rate (26.3 vs. 10.3%), suicidal plan rate (2.6 vs. 3.5%) and suicidal attempt rate (2.6 vs. 3.5%) between males and females, but smoking rate (0.0 vs. 65.5%) and married status were significant different (all *p* < 0.01). In addition, we found similar years of education, BMI and suicide risk distributions in both sexes, while the distribution of age was different. Only 7.9% of women were divorced, while only men admitted to be smoking. Up to 19.4% subjects reported severe suicidal ideation, with 26.3% for female and 10.3% for male.

In Table 2, we observe that total PANSS score (*F* = 5.843, df = 65, *p* < 0.05) and general psychopathology subscale (*F* = 8.166, df = 65, *p* < 0.01) were significantly higher in women than in men. This difference was still significant when adjusting for age, education, BMI and smoking (*p* < 0.05). No significant differences were observed in HAMD-24, HAMA, positive and negative subscales of PANSS between groups (all *p* > 0.05). As shown in Table 3, there was no significant difference in Memory quotient (MQ), verbal IQ, performance IQ, total IQ, reaction time (RT) of SCWT, digit sequencing and verbal fluency in BACS (all *p* > 0.05) when comparing men vs. women. However, BACS Composite score (*F* = 2.274, df = 65, *p* < 0.05), verbal memory (*F* = 5.877, df = 65, *p* < 0.01), token motor task (*F* = 2.989, df = 65, *p* < 0.01), symbol coding (*F* = 1.514, df = 65, *p* < 0.05), and tower of London (*F* = 10.659, df = 65, *p* < 0.01) in BACS were significantly higher in men than in women.

Table 4 shows levels of UA, HC, TC, TG and PRL in patients by sex. 10.4% (Female = 18.4% vs. Male = 0.0%) of patients had hyperhomocysteinemia; 7.5% (Female = 5.3% vs. Male = 10.3%) had hypertriglyceridemia and 11.9% (Female = 15.8% vs. Male = 6.9%) had hyperprolactinemia. There are significant differences in UA (*p* < 0.001), HC (*p* < 0.01) and TG (*p* < 0.05) between groups. These differences were still significant when adjusting for age, education, BMI and smoking (*p* < 0.05). Additionally, no significant differences were shown in levels of TC (*p* > 0.05) and PRL (*p* > 0.1).

**Table 4:** Mean baseline metabolic parameters of patients (Chengdu, China, 2018) by sex.

	**Female (*N* = 38)**	**Male (*N* = 29)**	***F***	**df**	***p***	**Total (*N* = 67)**
UA, mg/dL, M (S.D.)	**4.14 (0.82)**	**3.06 (0.66)**	**24.912**	**65**	**0.000**	**3.62 (0.92)**
Hypouricemia, *n* (%)	0 (0.0)	0 (0.0)				0 (0.0)
HC, μmol/L, M (S.D.)	**12.23 (15.86)**	**2.33 (4.11)**	**10.718**	**65**	**0.002**	**7.95 (13.14)**
Hyperhomocysteinemia, *n* (%)	**7 (18.4)**	0 (0.0)				**7 (10.4)**
TC, mg/dL, M (S.D.)	45.64 (20.61)	34.03 (28.87)	3.367	65	0.071	39.06 (26.10)
Hypercholesterolemia, *n* (%)	0 (0.0)	0 (0.0)				0 (0.0)
TG, mmol/L, M (S.D.)	**0.71 (0.75)**	**1.11 (0.79)**	**4.309**	**65**	**0.042**	**0.88 (0.78)**
Hypertriglyceridemia, *n* (%)	**2 (5.3)**	**3 (10.3)**				**5 (7.5)**
PRL, ng/mL, M (S.D.)	**22.17 (26.63)**	**10.61 (6.64)**	**3.206**	**65**	**0.082**	**16.69 (20.46)**
Hyperprolactinemia, *n* (%)	**6 (15.8)**	**2 (6.9)**				**8 (11.9)**

*UA, uric acid; HC, homocysteine; TC, total cholesterol; TG, triglyceride; PRL, prolactin; n, number; M, mean; S.D., standard deviation*.

*Values with significant sex differences are highlighted*.

## Discussion

Understanding the mechanisms underlying MAs before and during antipsychotic therapy of schizophrenia is very important due to practical implications in the management of these patients (56–58). In this report, our results indicate that FEDN patients already have metabolic changes before their antipsychotic treatment. Importantly, such phenomenon is more prevalent in women.

Accumulating evidence indicates that subthreshold metabolic dysregulation might be present in the premorbid phase of the illness (59) and in antipsychotic-naïve patients with first-episode psychosis (60), which has been confirmed in our report. We did not find hypercholesterolemia but hypertriglyceridemia in FEDN patients. In line with this finding, a recent meta-analysis by Frydecka et al. (2017) (9) revealed that patients with first-episode non-affective psychosis had significantly lower levels of TC as well as significantly higher levels of TG compared to controls. Lipid metabolic dysregulation observed in drug-naïve patients suggest that schizophrenia-spectrum disorders might share overlapping genetic background with cardio-metabolic phenotypes. Actually, the pathophysiology of schizophrenia seems to involve alterations in biosynthesis of TC, fatty acids (FAs), phospholipids (PLs) and sphingolipids (61–64). In addition, several neuropathological studies demonstrated alterations in the levels of cholesteryl esters (TC esters), TG, polyunsaturated FAs and PLs in prefrontal and frontal cortex of patients with schizophrenia (53).

Our results also showed that FEDN patients had higher prevalence of hyperhomocysteinemia and hyperprolactinemia. Previous studies have also shown significantly increased HC and PRL levels in antipsychotic-naïve schizophrenia and related disorders (11, 20). For example, Kirkpatrick et al. (13) reported significant differences in PRL levels in patients with schizophrenia compared with controls. Interestingly, HC can induce reactive oxygen species production, implicated in schizophrenia pathophysiology (65), by PAR-4 (protease-activated receptor-4) activation. Moreover, given that PRL is under negative control by dopamine (66), high level of PRL means dopamine dysfunction (67, 68). In this context, it is tempting to speculate that oxidative stress imbalance is one potential common pathway shared by MAs and schizophrenia. In addition, UA is a well-known antioxidant, so lower serum UA concentrations might be regarded as a peroxidation state (66). Although our sample did not show hypouricemia, UA level in the male was significant lower than that in the female, which is similar to the meta-analysis by Qiu He et al. (66).

To the best of our knowledge, this is the first report of sex differences in the frequency of hyperhomocysteinemia, hypertriglyceridemia and hyperprolactinemia in FEDN schizophrenia patients without medical comorbidities. We showed that nearly 10% of patients had metabolic disorders, independent of medical comorbidities and antipsychotic exposure. Some recent meta-analysis and case-control studies on antipsychotic-naïve patients with first-episode psychosis have found comparable results (14, 18, 69, 70). However, it is unclear whether these studies excluded subjects with medical comorbidities, especially type two diabetes, making complicated to rule out the confounding effects of medical comorbidities. For example, Yongjie Zhou et al. (14) reported that over 30% patients present with hypertriglyceridemia or hypercholesterolemia. They reported a much higher prevalence than we did, which might be due to the fact that they included FEDN patients with comorbid type II diabetes mellitus. In other words, what the previous studies with untreated patients with schizophrenia did not address is the extent to which the disease itself presents an increased risk for metabolic dysfunction independent of medical comorbidities. The results of our study showed that FEDN patients without medical comorbidities, especially women, were exceptionally vulnerable to metabolic abnormalities. The Clinical Antipsychotic Trials of Intervention Effectiveness (CATIE) study (71) found a higher prevalence for metabolic syndrome (MetS) in women (48.5%) than in men (42.6%). However, this study also recruited patients with antipsychotics that may affect the prevalence of MetS. A previous meta-analysis of the Chinese population showed that MetS was more common in women than in men (27.0 vs. 19.2 %) (72), which are consistent with our findings.

Our study has several limitations worthy of attention. First, although we shall recognize the heterogeneity that characterizes different regions of China (73), the Chengdu FEDN schizophrenia patients are able to typically represent the Han Chinese population. Second, our methods of measuring metabolite biomarkers were hypothesis driven and focused on assaying single metabolites, however, such high homogeneous and well-defined subject groups might be benefit from exploratory high-throughput metabolomic methods capable of quantifying hundreds of metabolites at one time (74). Third, current evidence suggests that the emergence of schizophrenia is associated with epigenetic characteristics (75). Genetic variation, such as microRNAs (17) and long noncoding RNAs (lncRNAs) (76), should be considered as another important parameter to be analyzed. Forth, environmental aspects during early stages of life, such as childhood trauma (77), and poor adult lifestyles (78) should be considered in future studies, which could contribute to the consistence of MAs and schizophrenia. Fifth, we did not include healthy controls for comparison, but definitions of hypouricemia, hyperhomocysteinemia, hypercholesterolemia, hypertriglyceridemia and hyperprolactinemia are based on reference values established for the Chinese population. Accordingly, the current results reflect the prevalence and sex differences of pathological MAs in subjects with schizophrenia. Last but not least, the sample size of patients we included was relatively small and the current study was limited to its cross-sectional design. However, in our next steps, we will integrate multiple dimensions (socio-demographics vs. psychopathological/cognitive vs. metabolic vs. neuroimaging), providing a comprehensive view on the MAs influencing psychiatric/cognitive performance in the early phases of schizophrenia. Due to the longitudinal nature of the study, we will eventually explore long term effects of metabolic abnormalities on the pathophysiology of schizophrenia.

## Conclusions

In conclusion, in this manuscript, we reported the baseline general characteristics of FEDN schizophrenia patients with a unique metabolic profile. A total of 67 patients were enrolled in this study, and 10.4%, 7.5%, and 11.9% of them were identified with hyperhomocysteinemia, hypertriglyceridemia and hyperprolactinemia, respectively. These results indicate that subclinical metabolic abnormalities may be present in patients with first episode schizophrenia.

## Data Availability Statement

The raw data supporting the conclusions of this article will be made available by the authors, without undue reservation.

## Ethics Statement

The studies involving human participants were reviewed and approved by the West China Hospital of Sichuan University and Chengdu Mental Health Center. The patients/participants provided their written informed consent to participate in this study.

## Author Contributions

JD and LJ completed the inclusion and exclusion of patients. LJ, QZ, XB, XZ, and YL evaluated the psychopathological symptoms. WC and LZ completed the blood sample test and EEG examination, respectively. QZ, HH, JD, and AT analyzed the data and wrote the paper. All authors contributed to the article and approved the submitted version.

## Conflict of Interest

The authors declare that the research was conducted in the absence of any commercial or financial relationships that could be construed as a potential conflict of interest.
